# Development of Flat Warts on the Cheeks after BioNTech-Pfizer BNT162b2 Vaccine: Is There a Correlation?

**DOI:** 10.3390/vaccines10040532

**Published:** 2022-03-29

**Authors:** Gerardo Cazzato, Paolo Romita, Caterina Foti, Debora Lobreglio, Irma Trilli, Anna Colagrande, Giuseppe Ingravallo, Leonardo Resta

**Affiliations:** 1 Section of Molecular Pathology, Department of Emergency and Organ Tranplantation (DETO), University of Bari “Aldo Moro”, 70124 Bari, Italy; anna.colagrande@gmail.com (A.C.); giuseppe.ingravallo@uniba.it (G.I.); leonardo.resta@uniba.it (L.R.); 2 Section of Dermatology and Venereology, Department of Biomedical Sciences and Oncology (DIMO), University of Bari “Aldo Moro”, 70124 Bari, Italy; paolo.romita@uniba.it (P.R.); caterina.foti@uniba.it (C.F.); debora.lobreglio@gmail.com (D.L.); 3 Odontomastotologic Clinic, University of Chieti “G. d’Annunzio”, 66100 Chieti, Italy; trilliirma@gmail.com

**Keywords:** flat warts, vaccines, cheeks, SARS-CoV-2, COVID-19, ADR

## Abstract

The SARS-CoV-2 pandemic has affected health systems across the globe, making the use of vaccines more urgent and topical than ever. Since the first months after the introduction of vaccinations, several reactions, both local and systemic, have been reported although they were mostly very mild and only rarely harbingers of more serious complications. We present a case of multiple flat warts onset over the cheeks in a patient after the second dose of mRNA BioNTech-Pfizer BNT162b2 vaccine, and we discuss the possible temporal association between the two events, also considering the patient’s antibody status.

## 1. Introduction

The severe acute respiratory syndrome coronavirus 2 (SARS-CoV-2) pandemic has affected the entire globe for more than two years [1], and only since the advent of vaccines has it been possible to partially return to normality [2]. The sudden circulation of the pandemic, the tight containment measures applied by governments and the more than significant rates of morbidity and mortality related to SARS-CoV-2 made it necessary to resort to different types of vaccination, and to implement the acceleration of mRNA vaccines trials [3,4,5,6,7,8]. Since the first months after the introduction of vaccinations, several reactions have been reported, both local and systemic [9,10], although these were mostly very mild reactions and only rarely harbingers of more serious complications [11]. Cutaneous adverse reactions were most commonly delayed large local reactions, followed by local injection-site reactions, urticarial eruptions, and morbilliform eruptions [12]. Less common reactions included pernio/chilblains, cosmetic filler reactions, zoster, herpes simplex flares, and pityriasis rosea-like reactions [12,13]. In this case report, we present a case of the appearance of numerous warty lesions after SARS-CoV-2 vaccination. To our knowledge, no such reaction has ever previously been reported, although it is not yet clear whether this is a purely chance association.

## 2. Case Presentation

**Figure 1:**
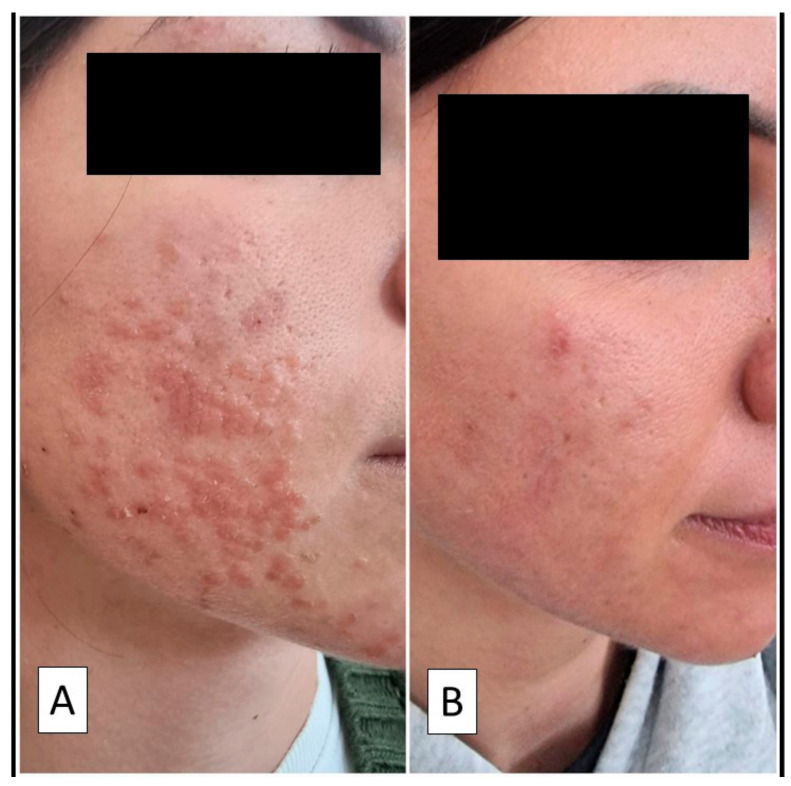
(**A**) Clinical photograph showing papular lesions, partly confluent in plaques, on the cheeks. (**B**) Almost complete remission of the lesions after two peeling sessions with 60% salicylic acid.

An atopic 28-year-old girl presented to the attention of the University Dermatology and Venerology Complex Operating Unit of BARI University Hospital, complaining of lesions on her cheeks that had developed over the last 7 days. The patient had come to our unit on her own initiative and was not in a premenstrual period. During the medical history, the patient declared that she had never suffered from flat warts and/or warty lesions in the past and had never undergone any aesthetic medicine procedures. She also stated that she had received the second dose of BioNTech-Pfizer BNT162b2 vaccine approximately one week earlier. After the administration of the first dose of the vaccine the patient declared that she did not develop any related symptoms. Blood counts and physico-chemical tests were normal. The range of c-reactive protein was around 0.50 mg/100 mL, while the erythrocyte sedimentation rate (ESR) settled at 18 mm/hour, indicating no ongoing inflammatory conditions. Polymerase chain reaction (PCR) of a nasopharyngeal molecular swab was negative, showing that the patient had never contracted COVID-19. Before coming to the dermatologist’s observation, the patient had decided to use a topical Tretinoin-based preparation, but without observing any benefit. The numerous lesions appeared as papules, partly confluent in plaques, with a verrucoid morphology (Figure 1A). In order to investigate the histological features of the skin lesions, a shaving biopsy of one of the verrucoid growths was performed.

**Figure 2:**
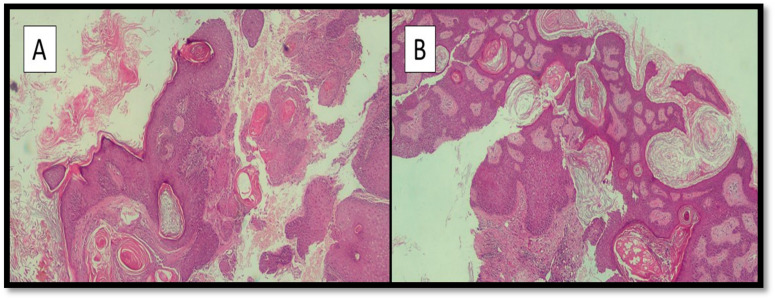
(**A**,**B**) Photomicrograph showing the histological characteristics of the lesions described in the main text: areas of acanthosis with hypergranulosis are noted (hematoxylin-eosin, original magnification 10×).

The sample was fixed in neutral formaldehyde buffered at 10% and sent to the Complex Operative Unit of Anatomy and Pathological Histology of the same hospital. After sampling, processing, microtome cutting and drying under a hood, preparations stained with hematoxylin/eosin were prepared and observed under an optical microscope. Histologically, a zone of acanthosis with hypergranulosis (Figure 2A) was evident, with a subtle, undulating surface change rather than the prominent finger-like papillomatosis of verruca vulgaris (Figure 2B). The morphological diagnosis of flat warts was confirmed.

After histological confirmation, the patient underwent two peeling sessions with 60% salicylic acid (performed two months after the onset of the lesions) and took an antiviral supplement with Echinacea for 3 months. There was no relapse at 6 months follow-up. Serological testing was positive for nonspecific IgM, positive for IgG (spike), and negative for IgG (nucleocapsid)—a pattern indicative of immunization after vaccination against SARS-CoV-2, as coding for the spike protein could be attributable to the mRNA contained in the vaccine.

## 3. Discussion

The SARS-CoV-2 pandemic has accelerated the approval of different types of vaccines, including both the consolidated modalities based upon viral vectors [14] and the most recent mRNA-based types [15]. Since the first weeks of vaccines delivery, different types of adverse reactions have been reported, mainly consisting of local reactions at the injection site [6,9,12]. For example, in their work, Català et al. [5] reported adverse reactions after vaccination with the vaccines NT162b2 (Pfizer-BioNTech; in 40.2% of the sample), mRNA-1273 (Moderna; in 36.3% of the sample) and AZD1222 (AstraZeneca; in 23.5%). Cutaneous reactions were classified as injection-site (‘COVID arm’, 32.1%), urticarial (14.6%), morbilliform (8.9%), papulovesicular (6.4%), pityriasis of rosea-like (4.9%) or purpuric type (4%). Varicella zoster and herpes simplex virus reactivations accounted for 13.8% of the reactions. The majority of skin lesions were self-limiting, except for 8 reactions (21%), classified as moderate/severe, which required special attention. McMahon et al. [12] reported their experience of 414 cutaneous reactions to mRNA COVID-19 vaccines, Moderna (83%) and Pfizer (17%), in the time period between December 2020 and February 2021. Delayed large local reactions were the most common, followed by local injection-site reactions, urticarial eruptions, and morbilliform eruptions. Less than half of the patients affected by first-dose reactions experienced a second-dose recurrence. Some other less common reactions included pernioerythema/chilblains, cosmetic filler reactions, zoster, herpes simplex flares, and pityriasis rosea-like reactions. In this presentation we describe the first reported case of multiple flat warts onset over the cheeks after the second dose of mRNA BioNTech-Pfizer BNT162b2 vaccine. After excluding any other acute skin disorders and/or any acute infections perhaps caused by some other types of microorganism on 3 PCR tests, and once ascertained the temporal association between the vaccination and the onset of the symptoms, we could hypothesize that this acute development of flat warts was most likely an adverse reaction to the BNT162b2 vaccine. This hypothesis was supported by the serological pattern, consistent with a post-vaccination immune reaction, so it seemed reasonable to postulate that this patient’s symptoms and signs were attributable to an adverse reaction.

Various authors have described a possible association between SARS-CoV-2 infection and the onset of autoimmune diseases via a mechanism involving molecular mimicry and cross-reaction. It is suggested, indeed, that these reactions may also be observed following vaccination, particularly in genetically predisposed individuals [16]. In fact, in his work, *Talotta* conducted an enzyme-linked immunosorbent assay (ELISA) analysis in which the anti-S1 glycoprotein monoclonal antibody and the antinucleocapsid of SARS-CoV-2 monoclonal antibody were incubated with 50 protein antigens, human tissues including, among others, transglutaminase 3 (tTG3), transglutaminase 2 (tTG2), ENA, myelin basic protein (MBP), mitochondria, nuclear antigen (NA), α-myosin, thyroid peroxidase (TPO), collagen, claudin 5 + 6 and S100B. The evidence provides a biological basis according to which vaccines inducing the synthesis of virus antigens can lead, at least theoretically, to the development of autoantibodies against certain antigens and, therefore, to the development of autoimmune diseases [16].

In this case of a patient with a past medical history of atopia, we hypothesize that the vaccine may have triggered an autoimmune reaction manifesting as the development of flat warts [17].

In the paper by Bellinato et al., in addition to a systematic review of the different types of lesions which could develop after the administration of SARS-CoV-2 vaccines, an attempt was made to address and investigate the possible immunopathological mechanisms responsible for determining these rashes, and it was concluded that the presence of local reactions at the injection site could be due to the presence of allergenic/immunogenic excipients such as polyethylene glycol-2000 (PEG) in the Pfizer/BioNTech vaccine and polysorbate 80 in the Oxford/AstraZeneca and Johnson vaccines [13,18]. As regards systemic reactions, although rare, they are also potentially life-threatening (for example severe cutaneous adverse reactions and therefore must be closely monitored and controlled [13,19].

## 4. Conclusions

In our case, it was not a serious reaction, but the association was based on a short time-frame in a patient who suffered from atopy. Therefore, it is a striking sign of the importance of extending our knowledge of these aspects, in order to establish any causal link and verify whether the temporal relationship is also based on a causative model.

## Data Availability

Not applicable.

## References

[1] Mistry P., Barmania F., Mellet J., Peta K., Strydom A., Viljoen I.M., James W., Gordon S., Pepper M.S. (2022). SARS-CoV-2 Var-iants, Vaccines, and Host Immunity. Front Immunol..

[2] To K.K., Sridhar S., Chiu K.H., Hung D.L., Li X., Hung I.F., Tam A.R., Chung T.W., Chan J.F., Zhang A.J. (2021). Lessons learned 1 year after SARS-CoV-2 emergence leading to COVID-19 pandemic. Emerg. Microbes Infect..

[3] Fiolet T., Kherabi Y., Macdonald C.-J., Ghosn J., Peiffer-Smadja N. (2022). Comparing COVID-19 Vaccines for Their Characteristics, Efficacy and Effectiveness against SARS-CoV-2 and Variants of Concern: A Narrative Review. Clin. Microbiol. Infect..

[4] Castro Dopico X., Ols S., Loré K., Karlsson Hedestam G.B. (2022). Immunity to SARS-CoV-2 induced by infection or vaccination. J. Intern. Med..

[5] Català A., Muñoz-Santos C., Galván-Casas C., Roncero I., Can M., Revilla Nebreda D., Solá-Truyols A., Giavedoni P., Llamas Velasco M., González-Cruz C. (2022). Cutaneous reactions after SARS-CoV-2 vaccination: A cross-sectional Spanish nationwide study of 405 cases. Br. J. Dermatol..

[6] Lipsitch M., Krammer F., Regev-Yochay G., Lustig Y., Balicer R.D. (2022). SARS-CoV-2 breakthrough infections in vaccinated in-dividuals: Measurement, causes and impact. Nat. Rev. Immunol..

[7] De Lucas Ramos P., García-Botella A., García-Lledó A., Gómez-Pavón J., González Del Castillo J., Hernández-Sampelayo T., Martín-Delgado M.C., Martín Sánchez F.J., Martínez-Sellés M., Molero García J.M. (2022). Actions and attitudes on the immunized patients against SARS-CoV-2. Rev. Esp. Quimioter..

[8] Fernandes Q., Inchakalody V.P., Merhi M., Mestiri S., Taib N., Moustafa Abo El-Ella D., Bedhiafi T., Raza A., Al-Zaidan L., Mohsen M.O. (2022). Emerging COVID-19 variants and their impact on SARS-CoV-2 diagnosis, therapeutics and vaccines. Ann. Med..

[9] Amanzio M., Mitsikostas D.D., Giovannelli F., Bartoli M., Cipriani G.E., Brown W.A. (2022). Adverse events of active and placebo groups in SARS-CoV-2 vaccine randomized trials: A systematic review. Lancet Reg. Health Eur..

[10] Walter E.B., Talaat K.R., Sabharwal C., Gurtman A., Lockhart S., Paulsen G.C., Barnett E.D., Muñoz F.M., Maldonado Y., Pahud B.A. (2022). Evaluation of the BNT162b2 COVID-19 Vaccine in Children 5 to 11 Years of Age. N. Engl. J. Med..

[11] García J.B., Ortega P.P., Fernández J.A.B., León A.C., Burgos L.R., Dorta E.C. (2021). Acute myocarditis after administration of the BNT162b2 vaccine against COVID-19. Rev. Esp. Cardiol..

[12] Mcmahon D.E., Amerson E., Rosenbach M., Lipoff J.B., Moustafa D., Tyagi A., Desai S.R., French L.E., Lim H.W., Thiers B.H. (2021). Cutaneous reactions reported after Moderna and Pfizer COVID-19 vaccination: A registry-based study of 414 cases. J. Am. Acad. Dermatol..

[13] Bellinato F., Maurelli M., Gisondi P., Girolomoni G. (2021). Cutaneous Adverse Reactions Associated with SARS-CoV-2 Vaccines. J. Clin. Med..

[14] Doerfler W. (2021). Adenoviral Vector DNA- and SARS-CoV-2 mRNA-Based COVID-19 Vaccines: Possible Integration into the Human Genome-Are Adenoviral Genes Expressed in Vector-based Vaccines?. Virus Res..

[15] Garcia-Beltran W.F., Denis K.J., Hoelzemer A., Lam E.C., Nitido A.D., Sheehan M.L., Berrios C., Ofoman O., Chang C.C., Hauser B.M. (2022). mRNA-based COVID-19 vaccine boosters induce neutralizing immunity against SARS-CoV-2 Omi-cron variant. Cell.

[16] Vojdani A., Kharrazian D. (2020). Potential antigenic cross-reactivity between SARS-CoV-2 and human tissue with a possible link to an increase in autoimmune diseases. Clin. Immunol..

[17] Lu Z., Zeng N., Cheng Y., Chen Y., Li Y., Lu Q., Xia Q., Luo D. (2021). Atopic dermatitis and risk of autoimmune diseases: A systematic review and meta-analysis. Allergy Asthma Clin. Immunol..

[18] Lederer K., Castaño D., Gómez Atria D., Oguin T.H., Wang S., Manzoni T.B., Muramatsu H., Hogan M.J., Amanat F., Cherubin P. (2020). SARS-CoV-2 mRNA Vaccines Foster Potent Antigen-Specific Germinal Center Responses Associated with Neutralizing Antibody Generation. Immunity.

[19] Kim M.A., Lee Y.W., Kim S.R., Kim J.H., Min T.K., Park H.S., Shin M., Ye Y.M., Lee S., Lee J. (2021). COVID-19 Vaccine-associated Anaphylaxis and Allergic Reactions: Consensus Statements of the KAAACI Urticaria/Angioedema/Anaphylaxis Working Group. Allergy Asthma Immunol. Res..

